# Review of Remarks on Professional Dental Education, Etc.

**Published:** 1852-07

**Authors:** A. Westcott

**Affiliations:** New York College of Dental Surgery


*A Review of Remarks on the Professional Education of Dentists, by John Trenor, Dentist, of New York.*—By Professor A. Westcott of the New York College of Dental Surgery, is just received, and from a hasty glance at its contents, we infer that Dr. Trenor will have to take up his pen again to maintain his position, and we see not how this is to be done if he does. For the benefit of the Medical Profession we have no objection to a Dental Chair in every Medical College, Such a chair well filled, would be of vast service to the practitioner of medicine. But to prepare young men for the practice of Dental Surgery in this way would be out of the question, and no one with a proper knowledge of the course in our Dental Schools, would think of such a thing.

In this review, Professor Westcott has handled the subject in a masterly manner.

